# MFP1/MSD-1 and MFP2/NSPH-2 co-localize with MSP during *C. elegans* spermatogenesis

**DOI:** 10.17912/micropub.biology.000427

**Published:** 2021-07-22

**Authors:** Kayleigh N. Morrison, Christopher M. Uyehara, James Matthew Ragle, Jordan D. Ward, Diane C. Shakes

**Affiliations:** 1 Department of Biology, William & Mary, Williamsburg, Virginia, USA; 2 Department of Molecular, Cell, and Developmental Biology, University of California – Santa Cruz, Santa Cruz, CA, USA

## Abstract

Until recently, the only verified component of Fibrous Bodies (FBs) within *Caenorhabditis elegans* spermatocytes was the Major Sperm Protein (MSP), a nematode-specific cytoskeletal element. Earlier studies in the pig parasite *Ascaris suum *had identified accessory proteins that facilitate MSP polymerization and depolymerization within the pseudopod of crawling spermatozoa. In this study, we show that *C. elegans* homologs of the two Ascaris accessory proteins MFP1 and MFP2 co-localize with MSP in both the pseudopods of *C. elegans* sperm and the FBs of *C. elegans* spermatocytes.

**Figure 1:**
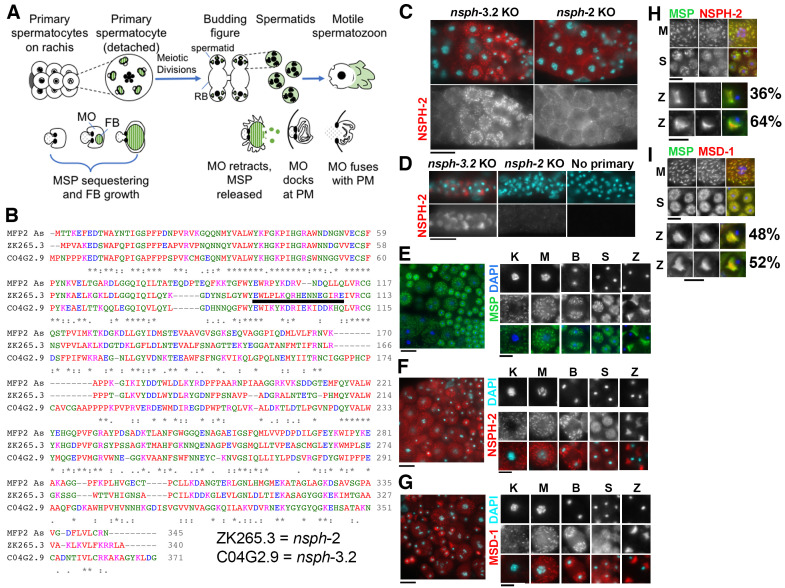
(A) Overview of spermatogenesis and fibrous body–membranous organelle (FB-MO) development. (Top) Spermatocytes develop on a syncytial rachis then detach to undergo the meiotic divisions. Following anaphase II, components which are no longer needed partition to a central residual body (RB). (Bottom) Within meiotic prophase spermatocytes, fibrous bodies (FBs) develop on the cytosolic face of the Golgi-derived membranous organelle (MO). During the budding division, FB-MO complexes partition to the spermatids. MOs then dock with the plasma membrane as the FBs disassemble and release MSP (Major Sperm Protein) dimers into the cytosol. During sperm activation, MSP localizes to the pseudopod, and MOs fuse with the plasma membrane. (B) Alignment of *C. elegans* ZK265.3/NSPH-2 (Nematode Specific Peptide family, group H) and C04G2.9/NSPH-3.2 with *Ascaris* MFP2 (MSP Fiber Protein) performed using the EMBL-EBI Clustal Omega Multiple Sequence Alignment tool (Madeira *et al.*, 2019). Peptide for the anti-NSPH-2 antibody is underlined. (C-D) DAPI and anti-NSPH-2 labelling in hermaphrodites with genetic knockouts of *nsph-3.2* and *nsph-2* respectively. (C) Left to right developmental sequence of developing spermatocytes and spermatids. (D) Hermaphrodite spermatozoa within spermatheca. No primary antibody controls were performed on *nsph-3.2* knockout animals. (E-G) Sperm spreads and individually staged cells with (E) anti-MSP labelling (n=63, 9 experiments), (F) anti-NSPH-2 labelling (n=50, 7 experiments), and (G) labelling against the MFP1 homolog, MSD-1 (Major Sperm protein Domain containing) (n=46, 3 experiments). (H-I) Co-labelling of MSP with either (H) anti-NSPH-2 or (I) anti-MSD-1. In spermatozoa (enlarged), the patterns were either fully (top) or partially overlapping (bottom). For NSPH-2/MSP n=16; for MSD-1/MSP n=14. Scale bars = 10 mm for C, D and E-G left; 5 mm for H, I and E-G right. Abbreviations: Karyosome spermatocytes (K), Metaphase spermatocytes (M), Budding Figures (B), Spermatids (S), and Spermatozoa (Z).

## Description

During the process of cell differentiation, specific cytoskeletal proteins can sequentially assemble into a wide variety of diverse molecular superstructures. Nematode spermatogenesis provides a powerful system for studying these transitions since sperm-specific transcription ceases prior to the meiotic divisions and translation ceases shortly thereafter (Chu and Shakes, 2013). Therefore, structural transitions that follow the meiotic divisions must be carried out by the remodeling of already synthesized proteins. The Major Sperm Protein (MSP) is a nematode-specific cytoskeletal element whose polymerization dynamics drive the pseudopod-based motility of the activated sperm (Roberts, 2005). In *C. elegans*, MSP additionally functions as the extracellular signaling molecule for triggering both ovulation and oocyte maturation (Miller *et al.*, 2003). MSP is highly abundant in sperm, where it reaches 10-15% of total and 40% of soluble cellular protein (Roberts 2005). Within developing spermatocytes, MSP is packaged into fibrous body–membranous organelle (FB-MO) complexes (Fig. 1A, Roberts *et al.*, 1986). By assembling into paracrystalline FBs, MSP is both sequestered away from the critical meiotic processes of chromosome segregation and cytokinesis while also being packaged for efficient segregation into spermatids during the post-meiotic partitioning process (Chu and Shakes 2013, Nishimura and L’Hernault, 2010, Price *et al.*, 2021). Following the meiotic divisions and sperm individualization, FBs disassemble, and MSP disperses as dimers throughout the spermatid cytoplasm (Fig. 1A). When sperm activate to form motile spermatozoa, MSP polymerization within the pseudopod drives the motility of the crawling sperm (Chu and Shakes, 2013). Thus, MSP exists in at least three distinct molecular states: 1) in highly organized paracrystalline FBs within developing spermatocytes 2) as unpolymerized dimers within spermatids, and 3) in dynamically polymerizing filaments and fibers within crawling spermatozoa.

Because MSP neither binds nucleotides nor assembles into polar filaments, its assembly and disassembly dynamics require accessory proteins. Previous biochemical studies in the pig parasite *Ascaris suum* identified MSP fiber proteins 1 and 2 (MFP1/2) as key regulators of MSP polymerization (Sepsenwol *et al.*, 1998, Buttery *et al.*, 2003, Grant *et al.,* 2005). MSP assembly dynamics within pseudopods requires the opposing forces of MFP1 and MFP2; MFP1 acts antagonistically to slow polymerization whereas phosphorylated MFP2 enhances MSP polymerization by serving as a nucleation site for polymer extension (Buttery *et al.*, 2003, Grant *et al.*, 2005). However, because these studies focused on MSP dynamics within crawling sperm and *in vitro* biochemical assays, the localization of these proteins within developing spermatocytes remained unknown. Would they be packaged along with MSP in FBs? Would the homologs of these proteins exhibit similar localization patterns during *C. elegans* spermatogenesis?

To confirm that the Ascaris proteins MFP2 and MFP1 have homologs in *C. elegans*, we compared protein sequences between the two species. We globally aligned Ascaris MFP2 with the predicted protein sequence of the two highest scoring *C. elegans* matches ZK265.3 and C04G2.9 (Fig. 1B). These are two spermatogenesis-expressed members of a larger MFP2 domain containing nematode specific peptide family, group H (Rödelsperger *et al.,* 2021), and will be hereafter referred to by their *C. elegans* gene prefix *nsph*. *Ascaris* MFP2 exhibits 51.4% identity and 66.7% sequence similarity with ZK265.3 (*nsph-2*) and 38.0% identity and 52.3% similarity with C04G2.9 (*nsph-3.2*). ZK265.3 and C04G2.9 have 41.0% identity and 52.6% sequence similarity with each other. Ascaris MFP1 has two isoforms, MFP1-a and MFP1-b, which when aligned with *C. elegans* MSD-1 (Major Sperm protein Domain containing), show 52% identity and 70.8% sequence similarity, and 51.9% identity and 66.0% sequence similarity, respectively (Buttery *et al.*, 2003). The sequence similarity between *A. suum* and *C. elegans* motility proteins suggests that they may have similar functions and binding properties.

To determine where NSPH-2 (MFP2) and MSD-1 (MFP1) localize within *C. elegans* spermatocytes and spermatozoa, we performed immunocytology experiments. For our analysis, we generated a rabbit polyclonal antibody against a unique NSPH-2 peptide (Fig. 1B) and obtained a polyclonal antibody against *C. elegans* MSD-1, from David Greenstein (Kosinski *et al.*, 2005). To test the specificity of the new anti-NSPH-2 antibody, we used high-efficiency genome editing to generate CRISPR-Cas9 knockout (KO) lines through insertion of multiple premature termination codons in all three frames for both ZK265.3 (*nsph-2*) and CO4G2.9 (*nsph-3.2*) (Wang *et al.*, 2018). Although neither knockout strain on its own exhibited a phenotype, they were useful for determining the specificity of our anti-NSPH-2 antibody. In control *nsph-3.2* KO hermaphrodites, the anti-NSPH-2 antibody labelled distinct structures within late-stage spermatocytes (Fig. 1C) as well as the pseudopods of spermatozoa within the spermatheca (Fig. 1D). In *nsph-2* KO hermaphrodites, only background labelling was observed. We then compared the localization of anti-NSPH-2 and anti-MSD-1 with the well characterized anti-MSP pattern in male gonads (Fig. 1E-G). In spermatozoa, both NSPH-2 and MSD-1 antibodies localize to the pseudopod, matching the known pattern in Ascaris spermatozoa. Within prophase and meiotically dividing spermatocytes, NSPH-2 and MSD-1 antibodies labelled discrete structures throughout the cytoplasm in a pattern strongly resembling FBs labelled by MSP antibodies (Fig. 1E-G). To verify that NSPH-2 and MSD-1 localized to FBs, we co-labelled male germlines with anti-MSP and either anti-MSD-1 or anti-NSPH-2 antibodies (Figure 1H-I). Within spermatocytes, the fully overlapping patterns revealed that NSPH-2 and MSD-1 are packaged along with MSP in the FBs. In spermatids, the MSP and MSD-1 were overlapping (Figure 1I). In contrast, except in immature spermatids that still retained their FBs, the NSPH-2 and MSP patterns only partially overlapped. In spermatids, the NSPH-2 labelling was notably less robust which may suggest that a change in protein conformation or binding partners is blocking the antigenic site. NSPH-2 does not appear to be degraded as it is present in the pseudopods of spermatozoa. All three antibodies labelled the pseudopods of spermatozoa, with the NSPH-2 and MSD-1 patterns either fully overlapping the MSP pattern (top set) or being restricted to a more central portion, adjacent to the cell body (bottom set).

Until recently, it was thought that Major Sperm Protein (MSP) was the only component of Fibrous Bodies (FBs) in *C. elegans*. Our discovery that MSD-1 and MFP2/NSPH-2 are packaged together with MSP in the FBs of developing spermatocytes supports a model in which the FBs function to gather, concentrate, and sequester proteins that will ultimately drive or regulate pseudopod motility. While early studies of MSP dynamics in Ascaris capitalized on its assets for biochemical approaches, parallel genetic approaches in *C. elegans* were stymied by the fact that many key factors, including MSP, MFP1 (MSD), and MFP2 (NSPH) are encoded by multigene families. Our own individual knockout strains of *nsph-2* and *nshp-3.*2 exhibit no obvious phenotypes. This result does not mean that MFP2/NSPH is non-essential, but that exploring its function may require knocking out combinations of up to five of non-identical, sperm-related NSPH genes. Similarly, the four MSD genes encode identical proteins that may need to be deleted to generate a mutant phenotype. CRISPR technologies give us the ability to knockout multiple genes and explore MSP dynamics in new ways. Beyond MSD and NSPH, will other proteins first identified in Ascaris as regulators of MSP dynamics and sperm motility (Buttery *et al.*, 2003) also localize to the FBs of developing spermatocytes? Alternatively, are there more proteins like the intrinsically disordered protein SPE-18 that localize to FBs and facilitate their growth and shaping but then are degraded shortly after the meiotic divisions (Price *et al.*, 2021)? Further studies will reveal whether MSD-1 and NSPH-2 function together with SPE-18 to facilitate FB assembly, or if they are just conveniently packaged in FBs alongside MSP.

## Methods

*Worm culture*

Worms were cultured on MYOB plates (Church *et al.*, 1995) and inoculated with the *E. coli* strain OP50, using methods similar to those described by Brenner (1974).

*Creation of knock-out lines*

CRISPR/Cas9 mutagenesis was performed as previously described (Paix *et al.*, 2014; Dokshin *et al.*, 2018; Wang *et al.*, 2018). Briefly, *C. elegans* strain N2 was gene-edited by the insertion of a 43-base-pair sequence that includes multiple stop codons in all three reading frames to disrupt translation (Wang *et al.*, 2018). A BamHI site was included 3’ to the stop codons to facilitate genotyping. Strains JDW307 *nsph-3.2(wrd56[C04G2.9::exon 1 STOP])* and JDW308 *nsph-2(wrd57[ZK265.3::exon 1 STOP])* were generated by CRISPR injection of an RNP complex [250 ng/µl of house-made Cas9 protein (Zuris *et al.*, 2015) , 50 ng/µl of the relevant crRNA, and 100 ng/µl tracrRNA] that was first incubated at 37°C for 15 minutes, then mixed with the co-injection marker pRF4 (50 ng/µl) and the relevant repair oligos (110 ng/µl) before being injected into wild-type L4 + 1 day worms grown at 20°C. After 4 days, rolling worms were plated individually, allowed to lay eggs, and the parental animal was genotyped by PCR and BamHI digestion. For candidate knock-ins the extrachromosomal array was removed by selecting non-roller progeny. Genotyping primer sequences can be provided upon request. The tracrRNA and crRNAs were obtained from Integrated DNA Technologies (IDT).

C04G2.9 crRNA: 5’-TACCGGGCTCGGCGGGAAGG-3’

ZK265.3 crRNA: 5’-AAAGGAAAACTGGATTTGGG-3’

*Repair oligos (BamHI sites in bold)*

C04G2.9/NSPH-3.2 AAGATACTTGGGCATTCCAGCCAATCGGAGCCCCCTGGGAAGTTTGTCCAGAGCAGAGGTGACTAAGTGATAA**GGATCC**TCCCGCCGAGCCCGGTAAAATGTATGGGTGAGCA

ZK265.3/NSPH-2

TACAACAAAGCGGAGTTGAAAGGAAAACTGGATTTGGGAAGTTTGTCCAGAGCAGAGGTGACTAAGTGATAA**GGATCC**GGGTGGACAGATTCAAATTCTTCAGTACAAGGGA

*Antibodies and immunocytochemistry*

Sperm spreads were obtained by dissecting 8-15 male worms per slide in 7.5 microliters of egg buffer (Edgar, 1995) on ColorFrost Plus slides (Fisher Scientific, 12-550) coated with poly-L-lysine (Sigma Aldrich, P8290). Light pressure was applied to coverslips to flatten the samples. Samples were then freeze cracked in liquid nitrogen and fixed overnight in −20°C methanol. Specimen preparation and antibody labeling followed established protocols (Shakes *et al.*, 2009). Primary antibodies included: 1:1500 4D5 mouse anti-MSP monoclonal (Kosinski *et al.*, 2005) and 1:200 rabbit anti-MSD-1 rabbit polyclonal (Kosinski *et al.*, 2005). Affinity purified rabbit antiserum against MFP2/NSPH-2 (ZK265.3) was generated by YenZym Antibodies using the peptide 92-108 EWLPLKQRHENNEGIRE. In experiments, the antibody was used at a 1:200 dilution. All samples were incubated with primary antibodies for 2 hours at room temperature. Affinity-purified secondary antibodies included 1:400 Alexa Fluor Plus 555 goat anti-rabbit IgG (Invitrogen, A32732) and 1:300 Alexa Fluor 488 goat anti-mouse IgG (H+L) (Jackson ImmunoResearch, 115-545-146). Final slides were mounted with Fluoro Gel with DABCO (Electron Microscopy Sciences #17985-02) containing DAPI.

*Imaging and analysis*

Images were acquired with epifluorescence using an Olympus BX60 microscope equipped with a QImaging EXi Aqua CCD camera. Photos were taken, merged, and exported for analysis using the program iVision. For control studies (Fig. 1C-D), image exposures were kept constant with no further image processing. For others, image exposures were optimized for individual gonads. The levels adjust function in Adobe Photoshop was used to spread the data containing regions of the image across the full range of tonalities.

## Reagents

**Table:** 

**Strain**	**Genotype**	**Available from**
N2	Wildtype *C. elegans* strain	CGC
CB4088	*him-5(e1490)* V	CGC
JDW307	*nsph-3.2(wrd56[C0G2.9::exon 1 STOP])*	J. Ward lab
JDW308	*nsph-2(wrd57[ZK265.3::exon 1 STOP])*	J. Ward lab
**Plasmid**	**Genotype**	**Description**
pRF4	co-injectable marker – dominant roller	4 kb fragment of genomic DNA from *rol-6(su1006)* collagen gene in Bluescript vector (Mello *et al.*, 1991).
**Antibody**	**Animal and clonality**	**Description**
anti-MSP	Mouse monoclonal, 4D5	Kosinski *et al.*, 2005
anti-MSD-1	Rabbit polyclonal, R194P	Kosinski *et al.*, 2005
anti-NSPH-2	Rabbit polyclonal	Rabbit antiserum was generated by immunizing a rabbit against the aa 92-108 (EWLPLKQRHENNEGIRE) of *C. elegans* ZK265.3, YenZym
Alexa Fluor Plus 555, secondary	Goat anti-rabbit IgG	Invitrogen, A32732
Alexa Fluor 488, secondary	Goat anti-mouse IgG	Jackson ImmunoResearch, 115-545-146
